# Hospital to Home: Supporting the Transition From Hospital to Home for Older Adults

**DOI:** 10.1177/08445621211044333

**Published:** 2021-10-27

**Authors:** Brittany Barber, Lori Weeks, Lexie Steeves-Dorey, Wendy McVeigh, Susan Stevens, Elaine Moody, Grace Warner

**Affiliations:** 1 Faculty of Health, 3688 Dalhousie University, Halifax, NS, Canada; 2 School of Nursing, 3688 Dalhousie University, Halifax, NS, Canada; 3 Rehabilitations & Supportive Care, 432234 Nova Scotia Health, Halifax, NS, Canada; 4 Continuing Care Central Zone, 432234 Nova Scotia Health, Halifax, NS, Canada; 5 Continuing Care, 432234 Nova Scotia Health, Halifax, NS, Canada; 6 School of Occupational Therapy, 3688 Dalhousie University, Halifax, NS, Canada

**Keywords:** Home care services, transitional care, acute care, long-term care homes, older adults, patient outcomes, health service delivery, home first

## Abstract

**Background:**

An increasing proportion of older adults experience avoidable hospitalizations, and some are potentially entering long-term care homes earlier and often unnecessarily. Older adults often lack adequate support to transition from hospital to home, without access to appropriate health services when they are needed in the community and resources to live safely at home.

**Purpose:**

This study collaborated with an existing enhanced home care program called Home Again in Nova Scotia, to identify factors that contribute to older adult patients being assessed as requiring long-term care when they could potentially return home with enhanced supports.

**Methods:**

Using a case study design, this study examined in-depth experiences of multiple stakeholders, from December 2019 to February 2020, through analysis of nine interviews for three focal patient cases including older adult patients, their family or friend caregivers, and healthcare professionals.

**Results:**

Findings indicate home care services for older adults are being sought too late, after hospital readmission, or a rapid decline in health status when family caregivers are already experiencing caregiver burnout. Limitations in home care services led to barriers preventing family caregivers from continuing to care for older adults at home.

**Conclusions:**

This study contributes knowledge about gaps within home care and transitional care services, highlighting the importance of investing in additional home care services for rehabilitation and prevention of rapidly deteriorating health.

## Background

The majority of older adults prefer to live at home and age in place for as long as possible (Canada Mortgage and Housing Corporation, 2020; Government of Canada, 2012), yet evidence indicates that older adults who are hospitalized are faced with numerous barriers to transitioning out of hospital and may be admitted to long-term care (LTC) homes unnecessarily (Di Pollina et al., 2017). LTC homes are intended for older adults that are not able to live independently in the community due to complex needs that require full-time assistance with activities of daily living and access to 24 hr nursing care (Canadian Institute for Health Information, 2021a). However, some older adults with mild or moderate health conditions or physical limitations may be entering LTC unnecessarily, instead of returning home with enhanced supports (Canadian Institute for Health Information, 2020a).

Within Canada, approximately one in nine newly admitted LTC residents could have potentially been cared for at home with added support (Canadian Institute for Health Information, 2020a). There are many factors that can contribute to older adults entering LTC homes earlier, such as difficulty navigating the health care system (e.g., being unaware of available home care services), financial barriers (e.g., significant out-of-pocket expenses and resources required to care for someone at home), health system responsiveness (e.g., finding reliable and consistent home care services), and access to specialized services (e.g., need for services tailored to specific geriatric health care needs and social or emotional support for family or friend caregivers) (Canadian Institute for Health Information, 2020b). Older adults that live in rural areas in comparison to urban areas are twice as likely to be admitted to LTC (Canadian Institute for Health Information, 2020a; Miller et al., 2014). Additionally, older adults that live alone compared to those living with family members are twice as likely to be admitted to LTC when they potentially could have been cared for at home (Canadian Institute for Health Information, 2020b; Miller et al., 2014).

### Challenges of Transitions for Older Adults

There are significant challenges that face older adults and their unpaid caregivers as they transition between healthcare settings, whereby continuity of care is disrupted (Hirschman et al., 2015; Weeks et al., 2018). Often, older adults and their caregivers are required to navigate within and between health settings and services to sustain their health, yet they experience inherent challenges in doing so, which are detrimental to their health outcomes and burden of acute care costs (Coleman et al., 2006). It is often during transitions between types of health services along the continuum of care that issues arise, such as long wait-times in hospital (McCloskey et al., 2015); poor communication of care needs and loss of information related to care plans (Reid et al., 2013; Schoen et al., 2011; Treiger & Lattimer, 2011); care that is delayed, unnecessary, not evidence-based, potentially unsafe, fragmented and poorly coordinated (Cummings et al., 2012; Reid et al., 2013); inappropriate placement in LTC facilities (Aschbrenner et al., 2011); exacerbated mental health issues (Aschbrenner et al., 2011); issues in accessing transportation (Aschbrenner et al., 2011); and additional burden placed on caregivers (Aschbrenner et al., 2011; McCloskey et al., 2015). There is a need to increase supports available for older adults and their caregivers, such as increasing uptake of home care services to reduce pressure on acute care hospital services and improve quality of care by ensuring older adults are receiving the most appropriate care, at the right time and place (Cummings et al., 2012; Reid et al., 2013; Robison et al., 2012).

Transitional care programs are designed to support older adults through the process of transitioning from hospital to home by providing discharge care planning, patient education, coordination and continuity of care, early identification and response to health changes, facilitating access to the right services at the right time, and planning for future health needs (Chalmers & Coleman, 2006; Coleman et al., 2006; Manderson et al., 2012; Weeks et al., 2016). When older adults and their caregivers do not receive adequate support from transitional care programs, they may lack access to appropriate services when they are needed, which can ultimately lead to an increased burden of care on acute care services and increased healthcare costs, such as earlier and unnecessary placement into residential LTC homes (Park et al., 2013; Watkins et al., 2012; Weeks et al., 2018).

### Reliance on Publicly Funded Acute Care Hospital Services

Acute care services in hospital are primarily designed to deliver short-term inpatient care and necessary treatment for a disease or severe episode of illness, with the goal to discharge patients as soon as they are medically stable (Canadian Institute for Health Information, 2021b). The purpose of acute care hospitalization is to address acute health conditions and acute exacerbation of chronic conditions (fall-related injuries, stroke, infection, surgical interventions). However, older adults often have a complex array of health considerations and are being admitted to acute care in hospital with multimorbidity and geriatric syndromes (e.g., frailty, impaired cognition, continence, gait and balance problems) (Chatterji et al., 2015; Stucki et al., 2018; Tijsen et al., 2019; Volpato et al., 2007; World Health Organization & World Bank, 2011). The increasingly complex health care needs of older adults mean they may require greater care and rehabilitation services focused on multiple conditions and support needs, which acute care settings do not typically provide during a hospitalization (Clarke et al., 2017; Tijsen et al., 2019). Rehabilitation within acute care hospital settings involves care from a multidisciplinary team (e.g., occupational therapists, physical therapists, psychologists, social workers, speech and language therapists, dietitians, nurses, and physicians) to evaluate, diagnose, and deliver therapeutic interventions for the purpose of restoring functional ability or enhancing residual capabilities for older adult patients (Medina-Mirapeix et al., 2017).

Rehabilitation is critical for older adults with multimorbidity and geriatric syndromes, given they have a higher risk for hospitalization and adverse outcomes, such as hospitalization-associated disability and inability to live independently upon discharge (Covinsky et al., 2011; Tijsen et al., 2019; Volpato et al., 2007). Rehabilitation has become increasingly important within acute care hospitalizations to address older adult complex conditions and has been found to have a positive impact on health outcomes such as improved functioning and mobility (Medina-Mirapeix et al., 2017), transition of patients from hospital to home (Medina-Mirapeix et al., 2017), reduced length of stay in hospital (Barnes et al., 2013), and reduced risk for nursing home admission (Barnes et al., 2013; Clarke et al., 2017). Unfortunately, there are wide health system discrepancies in the availability and delivery of rehabilitation for older adults within acute care hospital settings (Britton et al., 2015; Clarke et al., 2017), oftentimes resulting in a lack of meaningful activity and limited rehabilitative care for older adults with complex medical conditions during hospitalization (Boltz et al., 2010; Boyd et al., 2008; Clarke et al., 2017).

In addition to challenges of meeting complex health care needs of older adults during a hospitalization, there are immense economic burdens from high use of acute care hospital services (Roterman, 2017). A national study examining high-cost users of acute care hospital services indicated high hospital use among Canadians aged 50 or older was associated with extended length of stay before discharge to LTC (Roterman, 2017). Finding appropriate placement and moving older adults out of acute care hospital settings as soon as possible is ideal (Stucki et al., 2018; Tijsen et al., 2019; Volpato et al., 2007); not only to ensure complex health care needs can be met with appropriate rehabilitative services, but also to prevent worsening health conditions due to extended length of stay waiting to be discharged to LTC (Barnes et al., 2013; Roterman, 2017).

### Provision of Home Care Services

Health services used by older adults and their family or friend caregivers involve an array of public and private services that tend to operate in ‘silos’ independent of each other including home and community-based services, acute care, primary care, and social services (Hajek, 2013; Simpson, 2011). Within Canada, home health care services involve a range of public and privately funded organizations that provide skilled nursing services in the home, combined with a range of other home services such as personal care, housekeeping duties, physical therapy and rehabilitation, counselling, occupational and vocational therapy, dietary and nutritional services, speech therapy, audiology, medical equipment and supplies, medications, and intravenous therapy (Government of Canada, 2020).

The provision of home care services in Nova Scotia is funded and organized provincially between Department of Health and Wellness (i.e., governing public health policy), Nova Scotia Health (i.e., governing delivery of health services and programs), and delivered locally through Nova Scotia Health and Continuing Care coordinators that work with contracted agencies to provide home care services (Nova Scotia Department of Health and Wellness, 2020; Nova Scotia Health Authority, 2018). Home care services include skilled nursing care (i.e., dressing changes, catheter care, intravenous therapy, and palliative care), home support (i.e., personal care, respite, and essential housekeeping), and additional supports for caregivers (i.e., respite stay, hospital-type bed loan, specialized health equipment) (Nova Scotia Department of Health and Wellness, 2020; Nova Scotia Health Authority, 2018). There are no costs for receiving skilled nursing care services however, home support fees are based on income and family size, and additional supports for caregivers may include costs such as specialized equipment loans (Nova Scotia Department of Health and Wellness, 2020; Nova Scotia Health Authority, 2018).

Prior to the COVID-19 pandemic in the province of Nova Scotia, Canada, about 40–50% of patients staying in hospital for extended periods of time are deemed alternative level of care (ALC) due to the fact they are medically stable and no longer require acute medical care in hospital but are not discharged to return home (Nova Scotia Health Authority, 2017; 2019). The majority of patients that are assessed as ALC are older adults who are waiting in hospital for more appropriate settings such as placement in LTC (Gibbard, 2017; Nova Scotia Department of Health and Wellness, 2020). Existing research has identified that the needs of older adults could be met with home care, yet they are often unnecessarily placed into LTC due to unmet home care needs such as the need for physical assistance (Canadian Institute for Health Information, 2017; Hestevik et al., 2019; Statistics Canada, 2018; Wiercigroch & Weiyang, 2018). There is limited knowledge of the experiences of various stakeholders involved in the care of older adults, and older adults themselves, to investigate contextual factors involved in the decision-making process that indicate why older adult ALC patients in hospital are potentially inappropriately assessed as requiring LTC when they could return home with enhanced home care services. Although there are common issues and challenges that arise during transitions between types of health services (Aschbrenner et al., 2011; Cummings et al., 2012; McCloskey et al., 2015; Reid et al., 2013; Robison et al., 2012; Schoen et al., 2011; Treiger & Lattimer, 2011), it is critical that research identifies barriers to returning home for older adults including factors that are specific to local community contexts and acute care hospital settings.

### Understanding Gaps in Home Care Supports

Investments towards home care supports have recently been made in Nova Scotia through adoption of a Home First Philosophy, of which was first implemented in Ontario (Ontario Ministry of Health and Ministry of Long-Term Care, 2018), and applied to improve the development of Home Again enhanced services. The Home First Philosophy involves a shift in thinking where the focus is on giving older adult patients the chance to go home first with enhanced home services after an acute episode in hospital, instead of assuming a LTC home is the only option (Ontario Ministry of Health and Ministry of Long-Term Care, 2018). Home Again enhanced supports provide transitional care and additional hours of support or services beyond what is normally provided through the provincial government's home care program to facilitate a smooth transition home after a hospital admission (Canadian Institute for Health Information, 2017). Nova Scotia Health has identified challenges with current practices used to determine whether hospitalized patients will transition back to the community versus being placed in LTC. First, the current acute care assessment process may not appropriately identify hospitalized patients who would benefit from Home Again enhanced services. Second, additional time is needed to plan for early mobilization of patients while in hospital to optimize independence and functioning, and to organize critical community supports the patient needs post-discharge. Lastly, to ensure patients effectively transition from hospital to home, at least one family or friend caregiver needs to be involved throughout the whole process, so the needs of family or friend caregivers are met in the care plan. Oftentimes the unpaid family or friend caregivers provide significant care to patients transitioning home, making it critical to address their needs and resources within the care plan so they can continue providing care to patients at home.

The challenges identified by Nova Scotia Health indicate there is a need to understand how the gaps in home care services could be addressed to better meet patient and caregiver needs during the transition from hospital to home. Additional research is required to gather the experiences of older adult ALC patients and caregivers to identify how enhanced home care services could be adapted to support patients and their caregivers with the transition from hospital to home. The purpose of this study was to gain an in-depth understanding of characteristics and experiences of ALC patients, their family or friend caregivers, and health care professionals during an acute care hospital episode to identify factors influencing whether a patient returns home with enhanced home care services or is assessed as requiring LTC. These results contribute to understanding challenges and barriers facing ALC patients, factors influencing why they are potentially assessed prematurely for LTC placement, and how identified challenges and barriers could be addressed to support more older adults to return home with enhanced home care services.

## Methods

This qualitative study was conducted within an acute care unit at a hospital in Nova Scotia, where approximately one-third of the hospital's patient population was medically discharged older adults waiting for appropriate placement in LTC facilities. Research ethics approval for this study was obtained from Nova Scotia Health Research Ethics Board (#1024767) and we ensured ethical guidelines were met during the recruitment of patients, the informed consent processes, and maintaining confidentiality and protection of the data. We utilized a retrospective case study design to identify challenges and barriers influencing whether an older adult ALC patient returns home with enhanced home care services or is assessed as requiring LTC. A case study research design was chosen to support gathering in-depth understanding of lived experiences of patients and caregivers, as well as to investigate real-life contexts that influence decision-making processes (Crowe et al., 2011; Patton, 2002; Yin, 2006). Case studies are also effective for building evidence towards addressing an exploratory research question by gathering multiple perspectives of participant experiences (Hyett et al., 2014; Yin, 2006). A case was defined as a hospitalized older adult waiting for placement in LTC. Recruitment of each focal patient was challenging as it required a healthcare professional to identify patients that maintained some ability in performing activities of daily living and could potentially be discharged back home with enhanced community-based supports. Interviews were also conducted with family or friend caregivers and health professionals involved in the care of each focal participant to provide additional perspectives about the experiences of patients at home and contextual factors about assessing the patient for LTC once they were admitted to acute care in hospital.

The focal patients were selected for recruitment by a healthcare professional within the hospital from a list of all ALC patients waiting in hospital and assessed as requiring placement in LTC. We aimed to include a sample of three to four cases to generate a sample representative of ALC patients waiting in the chosen hospital study site at a given time. The research team could only complete three case studies and were in the process of starting to recruit a final patient case study in early months of 2020 at the start of the COVID-19 global pandemic. COVID-19 caused all research activities in hospital to end and prevented further recruitment of participants. Potential focal participants were purposely selected by prioritizing patients that were newly admitted to acute care in hospital, to reduce risk of attrition if a patient were to be transitioned out of hospital. The healthcare professional conducting recruitment initially contacted all potential participants following a set of inclusion criteria: patients assessed as requiring placement in LTC and were unable to return home to wait for placement; patients that spoke English; and patients with a family or friend caregiver who was willing and able to participate in an interview. If a patient could not provide informed consent, then the recruitment liaison contacted the designated substitute decision maker which was a family or friend caregivers. Both the patient or substitute decision maker and family or friend caregivers were asked whether they provide consent for healthcare providers to participate in an interview.

In-person interviews were conducted by the first author (BB) with the focus participant, one to two family or friend caregivers, and/or up to three health care professionals involved in providing care or making decisions about the future care of focus patient. A semi-structured interview guide was used during each interview to ensure data were systematically gathered to answer the overall research objectives. Interview questions focused on reasons the patient came to the hospital, main reasons the patient was not returning home, prior experience with home care services, what services or resources would be required for the patient to return home, and whether there was agreement or disagreement between family or caregivers and healthcare professionals for assessing the patient as requiring LTC. Case study interviews were audio recorded and transcribed verbatim. The interview data were analyzed by the first author (BB), principal investigator (LW), and a researcher assistant to identify themes and factors that emerged as influencing the inappropriate assessment of each patient for LTC placement. Data from interviews were analyzed for one patient, family or friend caregivers, and healthcare professionals in order to develop a comprehensive case. We used triangulation techniques to improve credibility and validity of findings by gathering insight for each patient case from multiple perspectives (i.e., patient, unpaid family caregiver, healthcare professional) (Carter et al., 2014; Patton, 2002; Yin, 2006). Participant names have been replaced with pseudonyms to protect identities of participants and confidentiality of patient information.

## Results

The findings will be presented by summarizing participant characteristics and then the findings from each case study for each focal participant.

### Participant Characteristics

Interviews with participants were conducted in person from November 2019 to the end of January 2020. We completed a total of nine interviews focused on three patient cases, two of which were female and one male including Mrs. Roy, Mrs. Jones, and Mr. Martin (see Table 1). Two additional patient cases were identified for recruitment. One case was excluded due to the patient dying in hospital before the researcher could conduct an interview with a family caregiver. A second patient case was excluded after the patient's family caregiver declined to participate in an interview for this study. We were required to gather consent from patients or their substitute decision maker, and family or friend caregivers, before we could interview healthcare professionals regarding the health and information of patients at the hospital. Only one patient participated in an interview, while two were precluded due to declining cognitive functioning. All participants were female (*n*  =  1 patient, *n*  =  4 family caregivers, and *n*  =  4 healthcare professionals).

**Table 1.** Case Study Participants.

	Case 1	Case 2	Case 3
Patient	Mrs. Roy Interview	Mrs. Jones No interview	Mr. martin No interview
Family or friend caregivers	Two daughters	Daughter	Spouse
Healthcare professionals	Nurse manager A Community care coordinator	Nurse manager B Nurse manager C	Nurse manager B Nurse manager C

### Case 1: Mrs. Roy

Mrs. Roy was living independently in her one-bedroom apartment that was also within the same building as her primary caregiver and daughter Sue. In her late 80s, Mrs. Roy experienced a slow decline in physical health due to heart disease and could no longer continue with activities that brought her joy, such as working as an editor at a local publisher. Due to mobility issues, Mrs. Roy began spending longer periods of time at home alone. Mrs. Roy's daughter Sue took on the responsibility of cooking most meals including breakfast and dinner as well as cleaning and assisting with some personal care when she had time before and after her workday at a fulltime job. There was little additional support from other family members to share in the responsibility of caring day-to-day for Mrs. Roy. Sue was responsible for providing and organizing support care for her mother, in addition to taking care of her own chronic health needs while working full-time. During the year prior to her admission, Mrs. Roy's physical strength began to deteriorate, and she could no longer take care of her own personal care needs such as getting on and off the toilet and bathing independently. As Mrs. Roy required help with daily activities, Sue acquired publicly funded home care services to come five days a week for 3 hr a day to assist with cleaning, cooking lunch, and personal care while she was at work.

As Mrs. Roy's health continued to deteriorate, she became immobile in a wheelchair and developed painful leg sores that required additional nursing care to clean and keep elevated. Sue advocated for increasing the amount and type of home care services by having home support come for an additional 2 hr a day to provide lunch, assist with toileting, and having nursing come to clean and dress her mother's leg sores. Mrs. Roy described her experience with home care as both positive and negative, such that she enjoyed socializing with some home support staff but felt frustrated that due to lack of time during her appointment some staff did not complete the services they were supposed to be providing like cleaning her apartment or her personal care. Sue, however, expressed overwhelming distress and concern that home support staff were not providing her mother with adequate personal care and she worried it may have contributed to the rapid deterioration in her mother's physical health in the form of a skin infection that developed and was not being treated adequately. Mrs. Roy's family was unaware of the severity of her skin infection and were devastated that neither home support staff nor nursing staff discussed these signs of deteriorating health.

In the autumn of 2019, Mrs. Roy was brought to hospital by ambulance due to renal failure and inability to control her bowels, at which point her family thought she was at the end of life. When Mrs. Roy was admitted to hospital, she was diagnosed with a skin infection on her legs and groin, as well as heart, liver and kidney failure. After spending 3 months in hospital Mrs. Roy's health began to stabilize and improve enough to be medically discharged from an acute care unit. Partly due to the family's negative experience with home care support and the primary care giver Sue experiencing burnout, Sue was not open to discussing with healthcare professionals the possibility of discharging her mother back home with enhanced home support. When Sue was asked about the potential of having enhanced home care services, she was unaware what the Home Again enhanced services would be able to provide above and beyond the home support and nursing services her mother was previously receiving. Sue also expressed concern about her mother's increasing frailty since being admitted to hospital, and a fear that because now her mother was accustomed to 24 hr care in hospital and she would not be able to regain enough strength or independence to return home without 24 hr support. There were high levels of family stress and financial strain in managing Mrs. Roy's apartment rent because her pension was being used to pay her ALC fees while in hospital as she waited for LTC. Part of the stress that Sue experienced was her uncertainty about how long her mother would have to wait in hospital for placement in a LTC home, the lack of control over choosing which home Mrs. Roy would be placed in, and lack of information about home care services earlier in the continuum of care.

The community care coordinator and nurse manager involved in Mrs. Roy's care described interactions with the patient and daughter as emotionally intense. The community care coordinator described challenges around communicating with the daughters about the processes within hospital and options available for their mother. Unfortunately, due to misinformation the daughters had previously received, they told healthcare professionals they believed their mother would be admitted to a LTC home of their preferred choice from hospital more quickly than if she stayed in community. As a result of prior negative experiences with home support and nursing care, the community care coordinator found it difficult to discuss the potential to transition Mrs. Roy home temporarily while waiting for a LTC home of their preferred choice and close in distance to their family neighborhood. The community care coordinator expressed sadness knowing some of the family's stress could be reduced if Mrs. Roy were transitioned home with enhanced home care services, such as reducing financial strain to pay for an ALC hospital bed or providing greater control over which LTC home Mrs. Roy would be placed in versus being admitted to the first LTC home with an available bed. The hospital nurse manager involved in Mrs. Roy's care described similar difficulties discussing options for transitioning Mrs. Roy out of hospital acute care with resources available through Home Again enhanced services. Due to the level of family distress and risk aversion to returning back home with enhanced home services, the daughters were unwilling to discuss any option other than LTC. When interviews for this case study were complete, Mrs. Roy had been waiting in hospital for 4 months and remained on the wait list for placement in a LTC home.

### Case 2: Mrs. Jones

Mrs. Jones was living independently in the lower level of her daughter's home for 6 years with her daughter Jane acting as the primary caregiver. When she was in her mid-90s, Mrs. Jones's physical health began to decline, and in the year prior to hospital admission, she required increasing amounts of care that her daughter Jane could not provide. Mrs. Jones experienced vision loss that prevented her from doing daily activities independently as well as leisure activities like crossword puzzles that would normally bring her joy. Jane advocated for publicly funded home care support for her mother to assist with personal care and meal preparation three times a week. As Mrs. Jones's health declined, she did not want to be home alone in the house and Jane retired early to provide more intensive 24hr care. There was no additional family support for Mrs. Jones's care and little spousal support to relieve Jane from primary caregiver duties. To relieve herself from the intense caregiving work, Jane was booking occasional weekend facility-based respite stays at a LTC home for her mother. Jane was receiving government funding through the “Supportive Care Program” that provided 500 dollars a month to assist with additional expenses incurred by caregivers.

In the summer of 2019 after a respite stay in a LTC home, Mrs. Jones contracted influenza and became bedridden and incontinent. Upon returning home with Jane, Mrs. Jones remained bedridden for 6 weeks at which point home support visits were increased to three times a day in the morning, afternoon and evening for 1 hr at a time. Overall, Jane's experience of having home services for her mother was positive; they were able to ease some of the demands that were required of Jane 24 hr a day. One of the biggest challenges of providing care at home was the inconsistent support staff that would come and go through the house. Jane was also unaware of how to address the absence of support services throughout the night, which became a significant problem when Mrs. Jones continuously called out for Jane throughout the night. Without any success trying prescription sleep medication for Mrs. Jones, the absence of overnight home care supports caused Jane to experience significant loss of sleep. After 6 weeks of sleep deprivation, Jane felt she had no other choice but to have her mother assessed for LTC and have her name placed on the wait list.

A few months later Mrs. Jones was taken by ambulance to hospital with pneumonia where she stayed for 7 weeks. Once Mrs. Jones was bedridden her health did not return to what it had been before, and she continued to deteriorate. Although healthcare staff tried to discuss transitioning her mother back home with enhanced home support, Jane felt as though the stress of being the only caregiver with little other support was too big a burden for her own mental and physical health, and LTC was the only viable option. When Mrs. Jones was medically discharged from hospital, she was transferred to a different hospital location where beds were allocated for patients requiring ALC and waiting for LTC. Part of the guilt that Jane experienced was being unable to provide the necessary care to her mother as a primary caregiver, as well as her lack of knowledge about navigating additional home care resources and services that could have provided rehabilitative care at home. Jane also felt powerless because she did not know how to plan ahead for her mother's care, such as placing her mother on a waitlist for LTC well in advance to a rapid deterioration in health and before she had to be admitted to hospital.

Two nurse managers that were involved in Mrs. Jones’ care recalled challenges that Jane faced, including the stress and fatigue from having to provide care throughout the night. Both nurse managers recognized that while Jane was accessing home support and nursing, she did not have adequate caregiver support and felt unable to continue being the sole caregiver at home. Nurse manager B described the difficulties of meeting the complex health care needs of older adults once they are admitted to acute care: there is a narrow timeframe available to rehabilitate patients and ensure they maintain their level of mobility and functioning. Unfortunately, after more than one acute care hospital stay Mrs. Jones’ health continued to decline and she was unable to receive the rehabilitative support required to regain strength or functioning. Nurse manager B felt a hospital acute care setting was not the best option for Mrs. Jones and it may have been more appropriate to intervene earlier to offer palliative enhanced home care services or transition to hospice. Nurse Manager C described the difficult position healthcare professionals find themselves in when trying to discuss options for getting patients back home when they have complex needs and are reliant on one caregiver, especially if the caregiver has already experienced burnout and is not willing to hear other options available to support them with caregiving duties at home. When interviews for this case study were complete, Mrs. Jones had been waiting in hospital for close to 3 months and remained on the wait list for placement in a LTC home.

### Case 3: Mr. Martin

Mr. Martin was living at home with his wife Maureen as his primary caregiver. Both Mr. and Mrs. Martin were in their mid-80s and primarily independent with an active lifestyle where they would go out every other day to local shopping malls, the casino for entertainment or various restaurants to eat. In the previous year, Mr. Martin suffered a stroke and was admitted to hospital for two and a half weeks for assessment of brain damage and additional health concerns. When Mr. Martin was discharged from hospital, he maintained some mobility using a walker but experienced overall decline in health that prevented him from participating in the same recreational activities as before. Maureen remained the sole primary caregiver with no additional support from family or friends and was responsible for taking care of all the cooking, cleaning, and Mr. Martin's personal care needs. The Martins had no prior experience with home care and were unaware of the options to have a home support worker assist with personal care or meal preparation. In the months leading to hospital admission Mr. Martin was receiving home visits from their family physician and a geriatrician to monitor a growth on his bowel. Maureen had been instructed by their family physician to bring her husband to hospital due to abdominal pain and concern about the increasing stress put upon Maureen as the primary caregiver in her 80s. Maureen planned on following through with their doctor's instruction to bring her husband to hospital at the end of the week however, in that same week Mr. Martin fell at home and was taken to hospital by ambulance.

Once Mr. Martin was admitted to hospital for his fall, he became immobile and experienced a rapid decline in cognitive health and was diagnosed with dementia and cancer. It was quickly evident to healthcare professionals that Maureen had been experiencing caregiver burnout and was no longer physically capable of providing care for Mr. Martin given his large stature; three healthcare professionals were required to lift and transfer him out of bed. Maureen was aware of home care services but was not interested in seeking services for herself or her husband due to hearing about her friends’ negative experiences and complaints about home care services.

Two nurse managers involved in Mr. Martin's care were concerned that Mr. Martin was not receiving any home care support and there was no record of referral to home care after Mr. Martin was discharged from his stroke. When the nurse managers asked Mrs. Martin about home care services, she had little knowledge of what home care services could have provided to assist with care after her husband's stroke. Both nurse manager B and C discussed the challenges associated with considering enhanced home care services as a viable option when there is only one primary caregiver and no additional support to relieve the caregiver from 24 hr duties. Although both nurse managers felt Mr. Martin could have potentially received enhanced home care services and remained safely at home, they felt it was not possible to discuss this option with Mrs. Martin due to her previous caregiver burnout, older age and health concerns, and lack of support to share in caregiving duties. Nurse managers B and C suggested it was difficult to ensure patients like Mr. Martin, who have complex needs and dementia, receive appropriate supports early on so that their health does not deteriorate as rapidly, and ultimately prepare for palliative home care services versus end-of-life care in an acute care hospital setting. Due to Mr. Martin's rapid deterioration of cognitive and physical health, he was unable to be stabilized enough to start the LTC admission process. During the last interview, nurse managers shared that Mr. Martin had died after spending 2 months in hospital.

## Discussion

In the context of one hospital and three case studies, results from this study indicate that information about home care services is not widely communicated across acute care health services. Family caregivers expressed they were hearing about the potential for enhanced home care services too late, after hospital readmission or a rapid decline in health status when family caregivers are already experiencing caregiver burnout. In each case study, family caregivers were experiencing extreme caregiver burnout as the sole caregiver and healthcare professionals felt it was very difficult to discuss the potential for patients to return home with enhanced home care services. Results indicate earlier access to enhanced home care services could have potentially reduced caregiver stress and prevented burnout for primary caregivers that have little to no additional support.

Existing evidence indicates that home care services can reduce the burden of acute care costs and bottlenecks in acute care hospital services by being implemented before older adults require acute care and recommending home care services upon discharge from their first hospital visit (Canadian Institute for Health Information, 2017; McGregor et al., 2018). In all three case studies, healthcare professionals described the challenge of discussing enhanced home care services for the first time with family caregivers when they were in a highly stressful situation in hospital, and after caregivers had expectations their loved ones would not be returning home. The challenge that healthcare professionals identified suggests conversations about enhanced home care services should be happening earlier along the continuum of care for older adults and their caregivers, so that caregivers have enough time to gather information about available home care services and can start receiving home care services to prevent a rapid decline in health. Findings from this study support existing evidence of the importance of connecting older adults with home care services earlier to help prevent rapid decline in health status, delay the onset of a major health event, and reduce the level of burnout and stress upon family or friend caregivers (Health Council of Canada, 2012; McGregor et al., 2018).

This study also contributes to existing knowledge of challenges to improve healthcare transitions for older adults and their family caregivers (Weeks et al., 2016), through identification of similar themes, like the need for information and education, and planning for future health needs of older adults. Providing adequate information and having conversations about home care services earlier in the continuum of care may also reduce misinformation about health system processes, such as caregiver expectations that acute care hospital services can be relied upon to get older adults into their preferred choice of LTC more quickly.

All three case studies revealed that there are limits to the services home care can provide, for example overnight home support. Canada's health care system is increasingly reliant on the role of the unpaid caregiver to support the needs of older adults however, many caregivers find themselves lacking resources or skills to cope with caregiving demands that are often 24 hr a day, 7 days a week (Canadian Public Health Association, 2016). Caregivers have indicated they could be better equipped to continue caring for older adults at home with improvements to the quality, type, and amount of formal home care services, funding for meal delivery services, and assistance navigating community-based respite supports (Canadian Institute for Health Information, 2020b). A reliance on unpaid caregivers indicates there is an increasing need to conduct caregiver assessments to fully understand unpaid caregiver needs and address how their needs could be met within a transitional care plan, so they are better equipped to continue caring for older adults at home.

It is important to explore the challenges older adults face living at home and the experiences of their unpaid caregivers in order to identify ways to ensure earlier uptake of home care resources. For instance, greater dissemination of information across health services describing what home care services and community resources are available to support older adults living safely at home across different local contexts such as urban versus rural settings. Greater dissemination of information about home care services could be achieved if healthcare providers facilitate conversations with patients across the continuum of care including primary care, geriatric services, emergency care, and acute care hospital services (Luker et al., 2019). Integrating healthcare professionals along the continuum of care for older adults, such as discharge planning nurses, acute care nurse case managers, nurse practitioners, or home care nurses, could potentially improve communication between health services and advocate for the health needs of older adults and their unpaid caregivers as they transition between health services (Allen et al., 2014; Thoma & Waite, 2018; Weeks et al., 2016). Healthcare professionals like nurse practitioners, discharge planning nurses, and home care nurses have valuable expertise and knowledge of health systems and understanding of the challenges older adults and their unpaid caregivers face once they transition home (Fels et al., 2015; Rhudy et al., 2009; Slatyer et al., 2019; Thoma & Waite, 2018).

The importance of an interprofessional health team for older adults across health services has been shown to improve health outcomes by accommodating for health needs with appropriate health services, delivering services for unpaid caregivers earlier so that older adults can live at home longer, and reduce health costs on acute care services (Allen et al., 2014; Buhr et al., 2019; Moore et al., 2012; Slatyer et al., 2019). Future research is required to identify strategies for providing information and education about navigating health system services such as home care services to older adults and their unpaid caregivers across a range of health services for older adults within primary health care and hospital settings (Buhr et al., 2019; Canadian Nurses Association, 2013; Moore et al., 2012). A health-systems approach would also enable a stronger understanding of gaps in service delivery and provision of government funds to improve care for older adults such as increasing the availability of overnight home support, rehabilitative services (e.g., occupational therapy, physiotherapy, social worker, dietician), community-based respite programs, medical equipment to improve quality of life at home, and direct funding programs for families to hire home care privately.

Results from this study highlight the difficult decisions families are faced with and the significance of systemic barriers—such as a lack of home care services and LTC beds—affecting the quality of life for older adults and their family caregivers (Nursing Homes of Nova Scotia Association, 2020). In each case study, the family caregivers did not have timely information about home care services and knowledge to navigate the resources required to continue caring for older adults at home. This finding contributes qualitative evidence to existing research, such as a recent meta-summary of older adult experiences after being discharged from hospital (Hestevik et al., 2019; Sun et al., 2017). Results of this meta-summary indicate older adults experienced a rushed transition out of hospital, insecurity due to lack of information about their diagnosis, and feeling unsafe due to uncertainty of ongoing care and self-care that is required at home (Hestevik et al., 2019; Sun et al., 2017). Evidence also indicates that although older adults are supported to return home after hospitalization, they are often dependent upon the support of an unpaid family or friend caregiver to overcome daily challenges with activities of daily living, such as cooking, dressing, and bathing (Hestevik et al., 2019).

Home care services are critical for supplementing care predominately provided by family or friend caregivers and becomes even more imperative for older adults with fewer social support networks of informal and unpaid caregivers to adapt to their different care needs within a home environment after returning home from being hospitalized. Older adults that live alone or do not have family or friend caregivers are often not able to return home from hospital and have no other place to go other than LTC (Wiercigroch & Weiyang, 2018). Older adults are also often financially vulnerable (Government of Canada, 2019) and face challenges of paying out-of-pocket for additional home care support above and beyond the publicly funded authorized hours (Penning et al., 2016), which covers up to 100 hr of home care support and up to 60 nursing visits in a 28-day period in Nova Scotia (Government of Canada, 2019; Nova Scotia Health Authority, 2019). When publicly funded home care services are not enough to support older adults to stay at home, hospitals become the catch-all of publicly funded health care services that family or friend caregivers and older adults rely on until they can transition to LTC (Kiran et al., 2020).

### Limitations

This study was intended to provide detailed accounts of the unique circumstances of three families and their experiences of health system challenges within one hospital setting. A limitation of a case study approach is that it may be difficult to transfer participant experiences to different hospital or community contexts. It is likely the experiences of patients, family or friend caregivers, and healthcare professionals vary across jurisdictions given the disproportionate availability of healthcare resources (such as home care services, rehabilitative services, or LTC homes), and access to acute care or primary care services for older adults and their family or friend caregivers. Additional research is required to understand the range of factors across different community settings that influence why older adults are prematurely assessed for LTC and not returning home with enhanced home care services. This includes the availability of home care services, capacity of hospital acute care services, and resources specifically designed to support unpaid family or friend caregivers so they can sustain a caregiving role. An additional limitation of this study was the unforeseen effects of the COVID-19 global pandemic that abruptly ended research activities in hospital and prevented additional recruitment of patient cases and data collection when this study was being conducted in early months of 2020.

### Implications for Future Research

Pressures upon LTC homes have become intensified by the COVID-19 global pandemic. In Canada, a major priority has been freeing up space in acute care hospital units to make room for the potential surge in COVID-19 patients, this has resulted in many older adults being discharged from acute care units, with some patients being sent to the first available bed in LTC (Canadian Medical Association Journal News, 2020). Older adults continue to be stuck in transition across Canada and forced to return home with inadequate home care supports and, in some cases, wait in hotels or community centers (Canadian Medical Association Journal News, 2020). COVID-19 has exposed many of the gaps and challenges that already exist within our health care system, and this research contributes important details from the narratives of patients, family caregivers, and healthcare professionals about systematic challenges within transitional care and home care services that will continue to persist.

A major challenge within Canada is in shifting health care resources towards prevention given our healthcare system is primarily designed to reactively address acute and episodic health issues for older adults with complex health conditions (Canadian Institute for Health Information, 2011; Health Council of Canada, 2010). Older adults with multiple and complex health conditions often experience worse health outcomes after an acute care hospitalization; however, they are less likely to receive the rehabilitative care they require once returning home in the community (Wang et al., 2019). Additional research is required to understand the availability and usage of home care services that support patients with complex needs and to identify the best place and point in time to connect older adults with home care services they require for health promotion, rehabilitation, and prevention of worsening health conditions leading to hospitalization (Wang et al., 2019). Additional research is also required to demonstrate how and why home care is an essential healthcare program and service, such as the impacts of implementing transitional care models (Hennessey & Suter, 2011; Hirschman et al., 2015; Weeks et al., 2020), the role of nursing healthcare professionals to support delivery of transitional care services (Camicia & Lutz, 2016; Hoplock et al., 2019; Kirby et al., 2014), and integration of discharge planning between health services so that appropriate home care services can be organized to support the needs of older adults and their unpaid caregivers transitioning from hospital to home (Goncalves-Bradley et al., 2016; McGilton et al., 2019; Naylor & Keating, 2009).

Demand for home care services will only continue to rise as the proportion of older adult populations continue to increase in many countries such as Canada (McGregor et al., 2018; Mitzner et al., 2009). In comparison to other countries, Canada expends a relatively modest budget for home care services. For example, of the 33-billion-dollar budget allocated for the provision of LTC in 2018, 82% was allocated to LTC facilities and only 18% was allocated to home care services (National Institute for Ageing, 2019). This is in contrast to the OECD country average of 35% of LTC budget on home care services (National Institute for Ageing, 2019). In Nova Scotia, only 19% of the total budget from the Provincial Department of Health and Wellness during 2019 was allocated to Continuing Care services which covers both LTC facility-based services and home care services (Nova Scotia Health Authority, 2019). Canada's relatively low investment in home care services does not reflect the growing need for home care services and the importance these community-based services have in preventative and rehabilitative care to reduce high costs across other healthcare services.

## Conclusions

Home care services are a critical piece of the public health system and continuum of care that can help to reduce costs, such as avoiding unnecessary hospitalization and improving the health and care of older adults by reducing the number of transitions between health services, hospital readmission, and premature admission to LTC. Now more than ever there is a need for investing in the availability and types of home care services across communities, which begins with understanding the challenges older adults face living at home and the experiences of their unpaid caregivers in order to identify ways to ensure earlier uptake of home care resources. Future improvements should prioritize increasing supports available for unpaid caregivers such as the number of service hours, access to a greater range of rehabilitative services, and to improve uptake of home care services earlier before a health decline occurs to ensure older adults have appropriate care and the choice to remain at home as long as possible.
